# Hybrid solder joints: the effect of nanosized ZrO_2_ particles on morphology of as-reflowed and thermally aged Sn–3.5Ag solder joints

**DOI:** 10.1007/s13204-023-02912-4

**Published:** 2023-07-07

**Authors:** Irina Wodak, Andriy Yakymovych, Peter Svec, Lubomir Orovcik, Golta Khatibi

**Affiliations:** 1 Institute of Chemical Technologies and Analytics, TU Wien, 1060 Vienna, Austria; 2 Christian Doppler Laboratory for Lifetime and Reliability of Interfaces in Complex Multi-Material Electronics, TU Wien, 1060 Vienna, Austria; 3 Department of Metal Physics, Institute of Physics, Slovak Academy of Sciences, Dubravska cesta 9, 84511 Bratislava, Slovakia; 4 Institute of Materials and Machine Mechanics, Slovak Academy of Sciences, Dubravska cesta 9, 84513 Bratislava, Slovakia

**Keywords:** Ceramic nanoparticles, Hybrid solder joint, Microstructure

## Abstract

The main number of current researches has been focused on the microstructure and mechanical properties of the Sn-based Sn–Ag–Cu-based solders, while various kinds of nanosized particles have been added. The synthesis and handling of ceramic nanosized powder are much easier than of metal nanoparticles. In addition, metal nanoparticles solved in solder joints during the soldering process or by thermal aging could behave as an alloying element similar to bulk metal additions, while ceramic nanoparticles retain their chemically inactive behavior in various thermal, thermo-mechanical, and electrical constraints. In some cases, the solved metal nanosized inclusions could increase the growth kinetics of the present intermetallic phases or even create new phases, which leads to more complexity in the predictions and simulations of chemical processes in the solder joints. Based on the assertions mentioned above, ceramic nanosized particles are industrially more favorable as reinforcing inclusions. On the other hand, there is no direct comparison in the literature between Sn-based Sn–Ag–Cu and Sn–Ag solder joints with similar ceramic nanoinclusions based on microstructural features and mechanical properties. In the present research, the Cu/flux + NPs/SAC/flux + NPs/Cu solder joints were produced with a nominal amount of 0.2 wt%, 0.5 wt%, and 1.0 wt% nanosized ZrO_2_ powder. The solder joints prepared via the above-described method are called in the literature as hybrid solder joints. The microstructure of the as-reflowed and thermally aged samples has been studied, especially at the interface solder/substrate. It has been shown that the minor additions of ZrO_2_ NPs lead to a decrease in the thickness of the Cu_6_Sn_5_ interfacial layer in the as-reflowed solder joints and a reduction in the growth kinetics of this layer, while the Cu_3_Sn interfacial IMC layer remains practically unaffected. Similar investigations were performed in our previous study but for both the hybrid and nanocomposite Sn–3.0Ag–0.5Cu solder joints. A comparative analysis of the impact of the ZrO_2_ nanoinclusions on the hybrid solder joints using Sn–3.5Ag and Sn–3.0Ag–0.5Cu has been performed.

## Introduction

One of the first main questions by research prearrangement of nanocomposite materials is related to the nature of the nanoparticles. For instance, weight, melting point, and chemical reactivity are the most important characteristics from the scientific point of view, while the possible difficulties considered by synthesis, storage, and handling are in focus for possible industrial implementation. Despite their different nature and interaction mechanisms, the various nanosized additions lead to the improvement of the microstructure in the bulk Sn–Ag–Cu (SAC) solder and at the interface between the solder and the substrate. For instance, it is expected that the ceramic nanoparticles change the morphology and the thickness of the interfacial Cu–Sn intermetallic (IMC) layer through the mechanical straightening effect. The metallic nanosized additions are expected to be dissolved either during the soldering process or subsequent thermal treatment. The atoms of the dissolved metal nanoparticles such as Co and Ni replace the Cu atoms in the interfacial Cu_6_Sn_5_ IMC layer. Furthermore, dissolved Co and Ni form Co(Ni)–Sn-based IMCs in the thermodynamically homogeneous solder alloy with increasing the concentration of these metal nanosized additions by more than1.0 wt% (Yakymovych et al. 2018; Yakymovych and Shtablavyi 2023). Based on the mentioned above, it is more complicated to simulate the microstructural changes in the solder joints doped with metal nanoparticles (NPs) under thermal and electrical constraints compared to the solder joints with minor additions of ceramic NPs.

There were several Sn-based alloys chosen as promising alternatives to Pb–Sn solders, namely Sn–Ag, Sn–Zn, and Sn–Ag–Cu alloys. In the last decade, the main focus of lead-free related studies was on SAC alloys, while a major number of these studies investigated the SAC solders doped with various types of nanoparticles. For instance, Pb-free Sn–Ag–Cu solders with minor additions of various ceramic nanosized particles and their joints have been widely studied (Al-sorory et al. 2022; Aspalter et al. 2020; Plevachuk et al. 2019; Wang et al. 2015; Yakymovych et al. 2016). The most frequently used nanosized ceramic powders are Al_2_O_3_, SiO_2_, TiO_2_, and ZrO_2_. It was postulated that the additions of ceramic nanoparticles suppressed the growth of the interfacial IMC layer due to the adsorption of NPs on the grain surface during solidification. For instance, it was reported that by adding 0.5 wt% and 1.0 wt% nano-ZrO_2_ into SAC305 solder paste the average thickness of the interfacial Cu_6_Sn_5_ IMC layer decreased, while the discontinuous scallop-type shape in the nanocomposite SAC305 solder joints was transformed to a more continuous scallop-type shape (Yakymovych et al. 2016). Similar results were observed by Aspalter et al. (2020) produced the hybrid solder joints as Cu/flux + nanoZrO_2_/SAC305/flux + nanoZrO_2_/Cu. The main difference between the nanocomposite and hybrid SAC solder joints is found to be the more pronounced impact of the nanoparticle additions in the hybrid solder joints. It should also be noted that a comparative analysis of the composite solder joints with nano-Al_2_O_3_, SiO_2_, TiO_2_, and ZrO_2_ showed the maximum impact of nanosized additions in the samples with nano-SiO_2_, while the impact of nano-SiO_2_ and nano-ZrO_2_ was comparable. There are only a few studies related to the impact of ceramic nanoparticles on the microstructure and mechanical properties of Sn–Ag solders. For instance, the ZrO_2_ nanoparticles added into the molten Sn-3.5Ag alloy have led to a refinement of the Ag3Sn intermetallic compounds (IMCs) formed during the solidification (Shen et al. 2006). These results were explained by the strong adsorption of the ceramic nanoparticles. The postulated increase in microhardness of the Sn–3.5Ag–nanoZrO_2_ samples was explained in the frame of the classic theory of dispersion strengthening.

In the present study, the commercial flux was mixed with the nanosized ZrO_2_ powder. The prepared nanocomposite flux was used to produce the solder joints, while their morphology and microstructure were studied afterward. A part of the samples was aged to investigate a thermal treatment effect.

## Materials and methods

The nanocomposite fluxes, denoted also as nanoemulsions, consisted of the commercial flux TACFlux 089HF (INDIUM Corporation) and the nanosized ZrO_2_ powder (TECNAN Navarrean Nanoproducts Technology) which were mechanically stirred for about 30 min at room temperature. The content of the added ceramic nanoparticles varied from 0.0 to 1.0 wt%. The characteristics of the commercial ceramic powders are presented in Table 1.

**Table 1:** Characteristics of the ceramic nanoparticles (TECNAN 2012)

Ceramic	Average particle size (nm)	Specific surface area (10^3^ m^2^ kg^−1^)
Nano-ZrO_2_	10–15	70–110

The bilateral solder joins were prepared using Cu plates and cylinders. The size of the substrates was 17 × 10 × 2 mm, the cylinders on top of the samples were 4 × 4 mm (diameter and height), and the thickness of the Sn–3.5Ag foil was 0.2 mm.

**Fig. 1:**
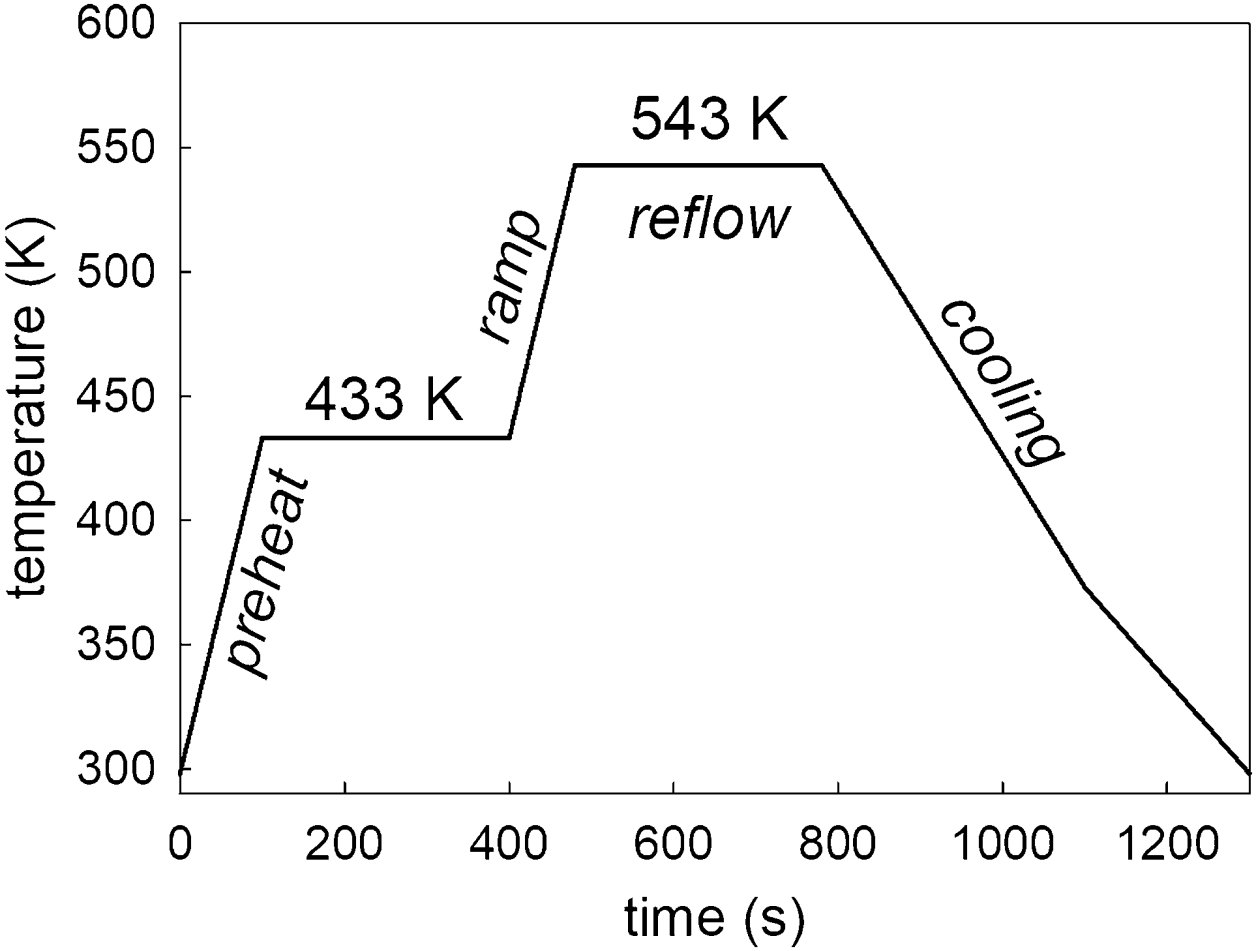
Reflow temperature profile of hybrid solder joint

The soldering process was conducted with a reflow oven according to the reflow temperature profile presented in Fig. 1. The joint samples were polished afterward to remove the extruded solder and flux residue from the edges. A part of the as-reflowed samples was exposed to a heat treatment at 453 K for 96, 192, and 288 h, respectively.

A JEOL JSM-7600F is a field-emission scanning electron microscope equipped with energy-dispersive X-ray analyzer (EDX), which was used for the microstructural analysis of the interfacial phases between the solder and the Cu substrate. High-resolution images were obtained by the backscattered electrons (BSE) employing the electron beam with 15–20 kV excitation energy. The ImageJ software which is freely available on the Internet (https://imagej.net/ij/index.html) was used for the estimation of the average thickness of the interfacial IMC layer.

## Results and discussion

### Morphology analysis and formation of the interfacial IMCs layer

The ceramic nanoparticles are chemically non-reactive in the solder joints. They do not form any chemical compounds neither with the solder nor with the substrate during the soldering process. Therefore, it is extremely challenging to find their traces in the solder joints by means of EDX analysis. One of the most popular indirect methods to indicate that the main part of the ceramic NPs remains in the solder is to inspect the morphology and microstructure of the part on the solder joints where the highest concentration of the nanosized inclusions is expected. In the present study, the ceramic nanoparticles mixed with the flux to reach the maximal concentration of those between the solder and substrate. According to the theory of adsorption of a surface-active material, the ceramic nanoparticles should maximize the number of adsorbed particles on the surface of the intermetallic compounds decreasing their surface energy and growth rate (Yakymovych et al. 2016). Therefore, the discontinuous scallop-type shape of the Cu_6_Sn_5_ interface IMC layer between the solder and substrate, which is common for the SAC and SA solder joints, should change into a more planar-type shape.

**Fig. 2:**
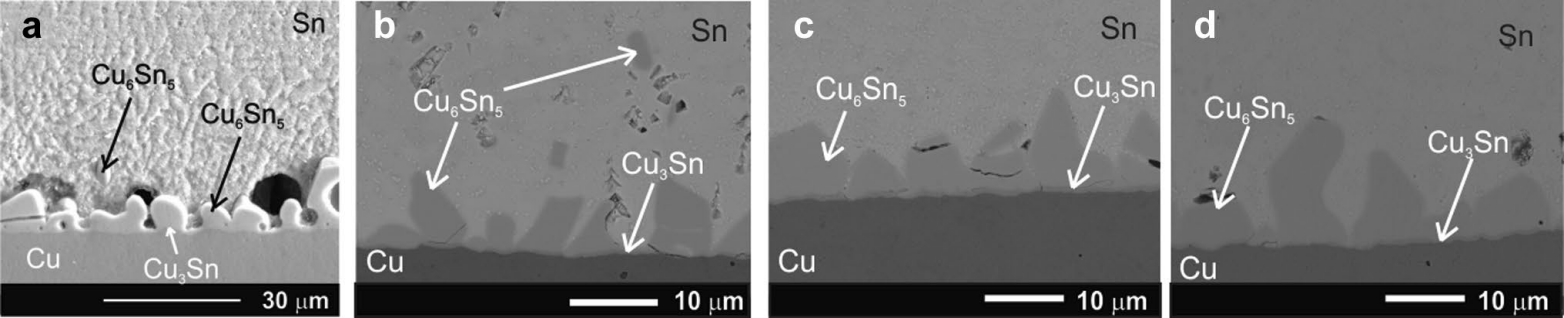
SEM micrographs of the as-solidified Cu/nanocomposite flux/Sn–3.5Ag/nanocomposite flux/Cu hybrid solder joints with 0.0 wt% (**a**), 0.2 wt% nano-ZrO_2_ (**b**), 0.5 wt% nano-ZrO_2_ (**c**), and 1.0 wt% nano-ZrO_2_ (**d**)

**Fig. 3:**
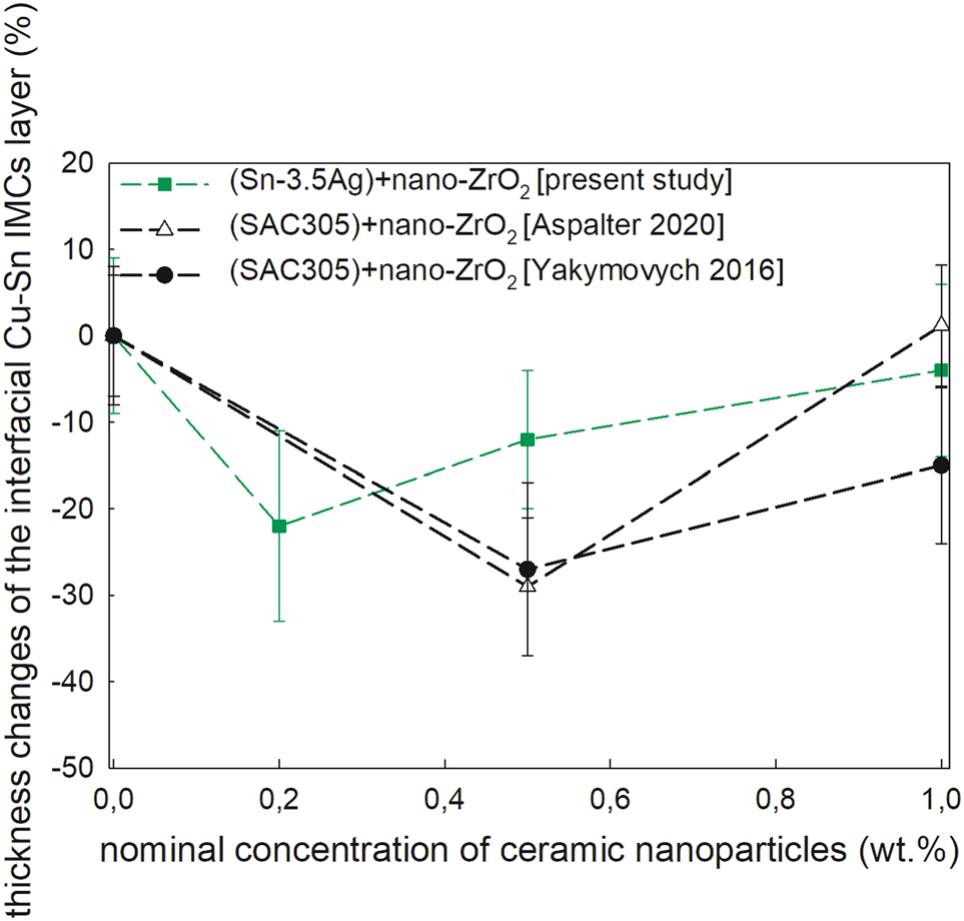
Thickness changes of the interfacial (Cu, Ni)–Sn IMCs layer in the as-solidified solder joints with nanosized ceramic inclusions

The SEM micrographs and the corresponding EDX analysis of the as-solidified samples show that the interfacial Cu_6_Sn_5_ grains, which are extended into the solder matrix, become to be smaller for the joints with nanosized ceramic additions (Fig. 2a–g). Addition of the nanoparticles as adsorption elements changes the driving force for the growth of the interfacial IMCs to the more planar direction and decreases the surface energy of the IMCs layer which leads to a reduction of the growth velocity of that plane. The morphology of the interfacial Cu_6_Sn_5_ layer has shown a change from scallop-like to prism-like for the samples with ZrO_2_ nanoinclusions (Fig. 2b–d). A very thin and planar Cu_3_Sn IMC layer is detected between the Cu_6_Sn_5_ IMC layer and the Cu substrate similar to our current study of the hybrid Sn–3.5Ag solder joints with Ni NPs. This IMC layer observed by conventional SEM is almost undistinguishable from the unaided eye due to a very thin Cu_3_Sn IMC. That could be a reason, why it was not detected in the as-reflowed hybrid SAC305 solder joint with nano-sized Al_2_O_3_, SiO_2_, TiO_2_, and ZrO_2_ (Aspalter et al. 2020). The changes in the thickness of the interfacial IMCs layer in the as-solidified hybrid Sn–3.5Ag solder joints are presented in Fig. 3 in percentage to compare it with the literature data both for the nanocomposite (Yakymovych et al. 2016) and hybrid SAC305 solder joints (Aspalter et al. 2020). The thickness of the interfacial Cu_6_Sn_5_/Cu_3_Sn layer in the Sn–3.5Ag solder joint equals 5.60 µm, 4.60 µm in the nanocomposite SAC305 solder joint, and 3.30 µm in the hybrid SAC305 solder joint.

The main purpose of Fig. 3 is to establish if we have a similar tendency by adding ceramic NPs using two different approaches of introducing the ZrO_2_ NPs additions into the solder joint, namely either via mixing with solder paste or with flux placed between solder and substrate. It shows that the thickness of the IMCs layer decreased in both cases, while the maximal effect was reached at lower concentrations by using the nanocomposite flux + solder foil compared to the nanocomposite Sn–3.5Ag solder joints. This effect is less pronounced in the joints with the higher amount of the ZrO_2_ NPs additions due to their agglomeration and segregation resulting in a decrease in the surface energy and a reduction in the number of the surface-active nanoparticles. It is hardly possible to perform the qualitative comparison due to a lack of additional information such as the proportion of the nanoinclusions concentrated at the interface solder/substrate. However, it was also expected that the present method should localize the most amount of those in this area. The interfacial Cu_3_Sn layer is practically not affected by additions of the ceramic nanoinclusions.

**Fig. 4:**
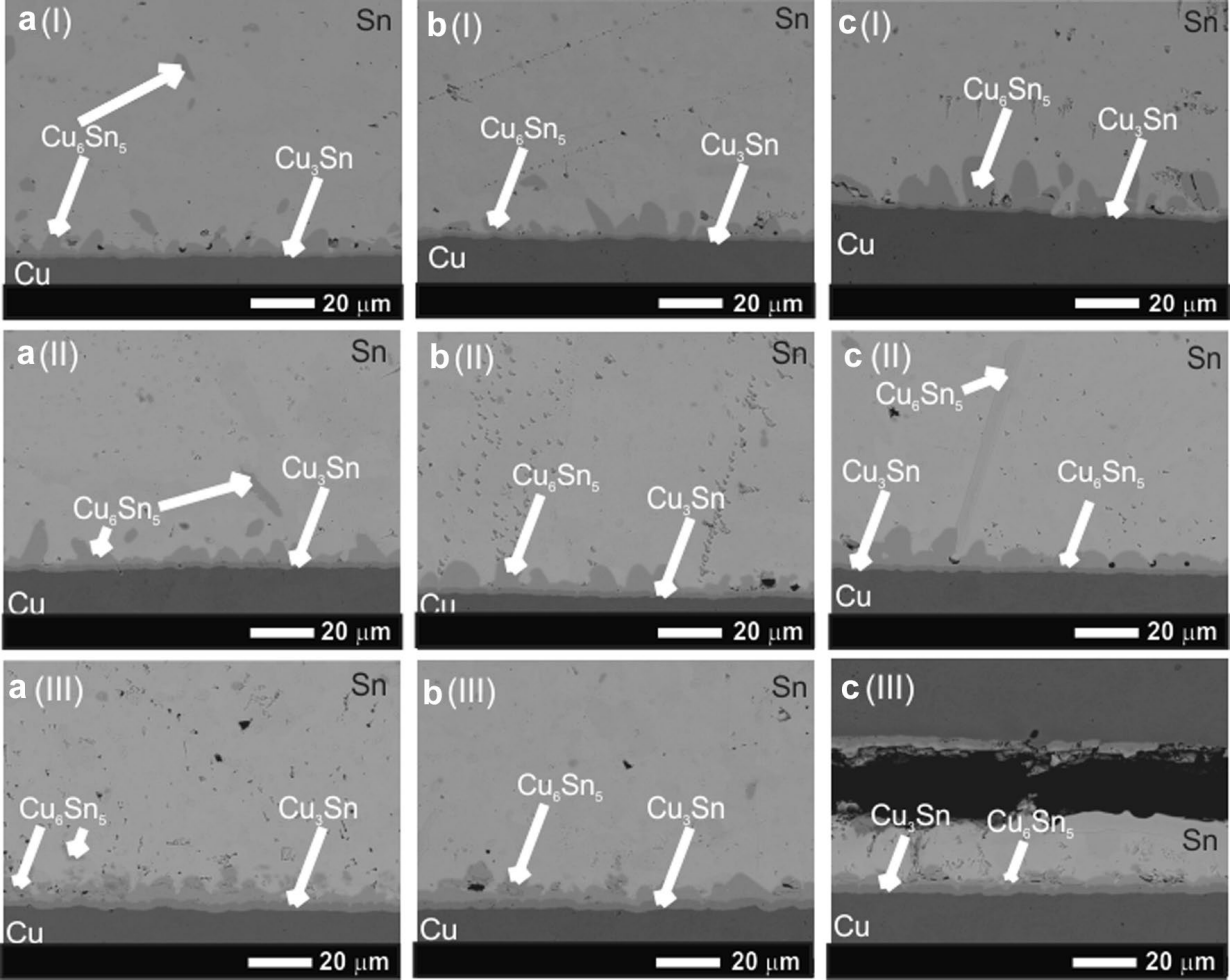
SEM micrographs of the thermal aged Cu/nanocomposite flux/Sn–3.5Ag/nanocomposite flux/Cu hybrid solder joints with 0.2 wt% (**a**), 0.5 wt% (**b**), and 1.0 wt% (**c**) of nanosized ZrO_2_ particles at 423 K for 96 h (I), 192 h (II), and 312 h (III)

A part of the samples was thermally aged at the temperature of 453 K for 96, 192, and 312 h, respectively (Fig. 4).

The continuous scallop-type shape of the interfacial IMC layer obtained in the as-reflowed samples was transformed to a more planar shape with increased aging time and increased amount of the nanoinclusions up to 1 wt%. It should be also noted that the thickness of not only Cu_6_Sn_5_ IMC but also of the Cu_3_Sn IMC was significantly increased during the aging and remained the flat-type shape.

### Growth kinetics of interfacial IMCs

There are several works related to the analysis of such changes in growth kinetics based on empirical kinetic laws. It is expected that the interfacial IMCs layers grow continuously during isothermal aging by the following reactions:

$$6{\text{Cu}} + 5{\text{Sn}} \to {\text{Cu}}_{6} {\text{Sn}}_{5} ;$$

$${\text{Cu}}_{{6}} {\text{Sn}}_{5} + 9{\text{Cu}} \to 5{\text{Cu}}3{\text{Sn}};$$

$$3{\text{Cu}} + {\text{Sn}} \to {\text{Cu}}_{3} {\text{Sn}}.$$

Arafat et al. (2020) have modeled intermetallic growth between Cu and Sn aged at different temperatures. It is supposed that the much slower growth rate of the interfacial IMCs in the aged solder joints at temperatures lower than 373 K, and especially of Cu_3_Sn, is mainly related to the substantially slower diffusion of Cu atoms to the IMC growth fronts at these temperatures.

**Fig. 5:**
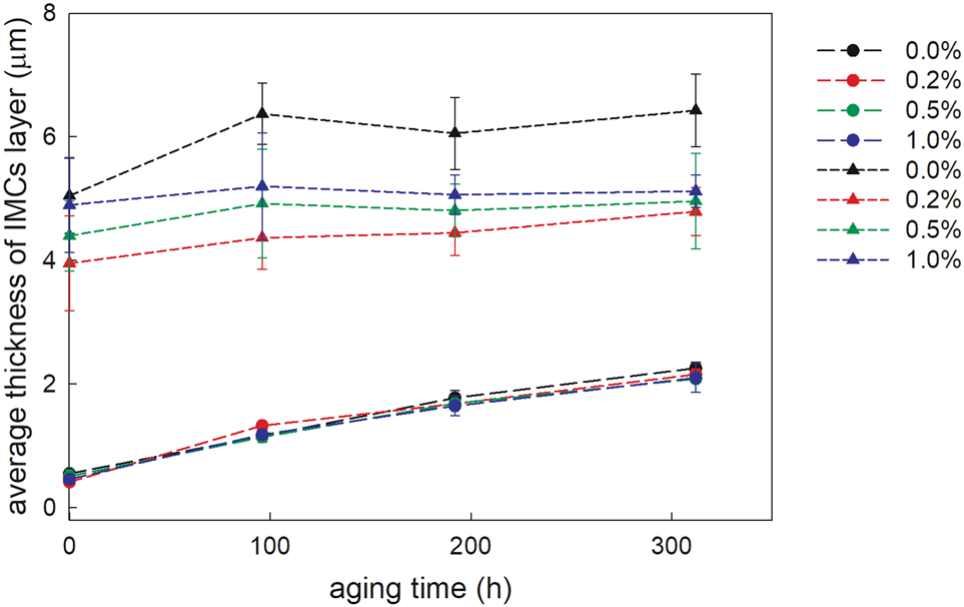
The average thickness of the interfacial Cu_6_Sn_5_ (triangles) and Cu_3_Sn (circles) IMCs in the thermally aged samples at 453 K

The Cu/Ag3.5–Sn/Cu solder joints thermal aged at 453 K for 96 h show a proportional increase in the thickness of both interfacial Cu–Sn IMCs. Further aging has led to much slower growth of the interfacial Cu_6_Sn_5_ layer even being practically constant in the time period between about 100 h and 200 h (Fig. 5). A decrease in the growth rate of the interfacial Cu_6_Sn_5_/Cu_3_Sn layer in the hybrid SAC305 solder joints with minor nanosized ZrO_2_ inclusions was indicated after 137 h of the isothermal aging at 453 K by Aspalter et al. (2020). At that time, the growth rate of the interfacial Cu_3_Sn layer remained constant for the entire time period. Ajay Kumar and Dutta (2018) pointed out that the interfacial Cu_3_Sn grows mainly by consumption of the Cu substrate and Cu_6_Sn_5_ IMC layer, while the Cu_6_Sn_5_ grows essentially at the Sn/Cu_6_Sn_5_ interface and to a lesser extent at the Cu_3_Sn/Cu_6_Sn_5_ interface. This tendency is stronger pronounced in the solder joints doped with ZrO_2_ because ceramic nanosized inclusions decrease the growth kinetics of the Cu_6_Sn_5_ IMCs. In contrast, the growth rate of the Cu_3_Sn IMC remains to be similar to the undoped solder joint.

**Fig. 6:**
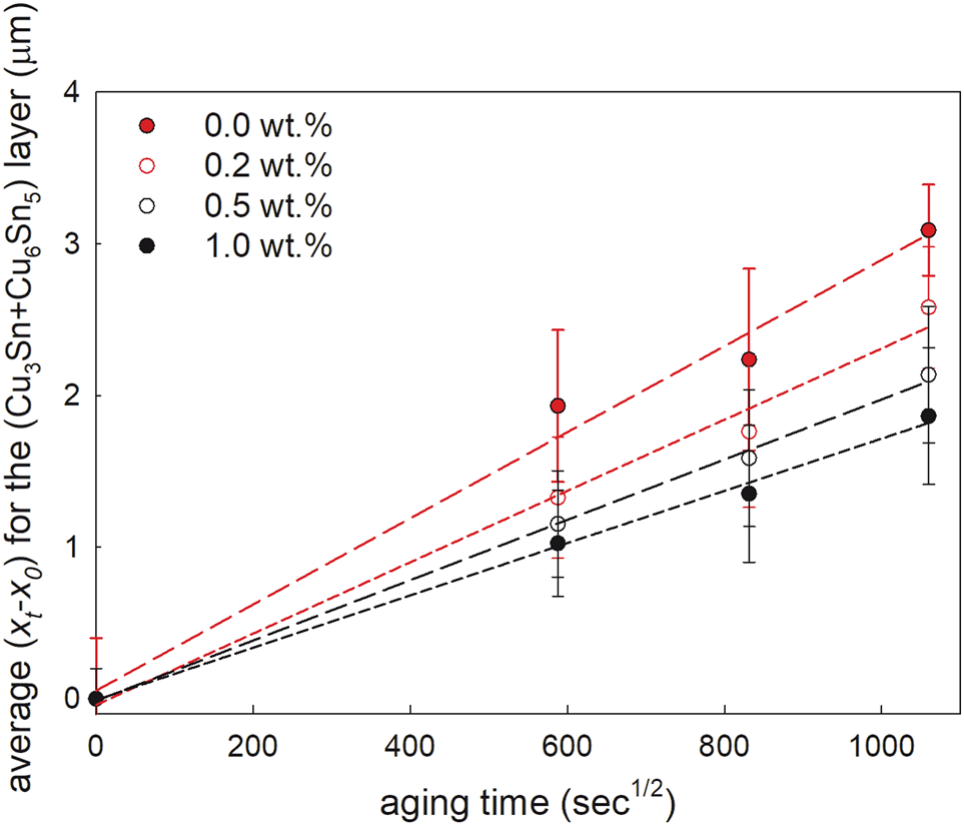
The aging time dependence of the average thickness of the interfacial IMCs layer in the aged solder joints at 453 K

The relationship between the IMC layer thickness and aging time can be expressed by the following equation:

1 $$\left( {x_{t} - x_{0} } \right) = (D_{{{\text{eff}}}} t)^{1/2}$$

where *x*_0_ is the IMC thickness of the as-solidified joint and *x*_*t*_ is the IMC thickness at aging time *t*. The effective diffusion coefficient of the IMC during thermal aging *D*_eff_ can be determined from the slope of the linear dependence between the IMC thickness, (*x*_0 − _*x*_*t*_), and the square root of the aging time, *t*^1/2^ (Fig. 6):

2 $$\left( {x_{t} - x_{0} } \right) = a + b \times t^{1/2}$$

The calculated parameter *b* of Eq. (2) and the effective diffusion coefficient are presented in Table 2.

**Table 2:** Parameter *b* of linear regression (2) and the effective diffusion coefficient for the interfacial Cu–Sn IMCs layer of the solder joints aged at 453 K

wt% of ZrO_2_ in the flux	0.0	0.2	0.5	1.0
*b*, µm s^−1/2^	0.0028 ± 0.0003	0.0024 ± 0.0002	0.0020 ± 0.0001	0.0017 ± 0.0001
*D*_eff_, 10^–18^ m^2^ s^−1^	7.84 ± 0.01	5.76 ± 0.01	4.00 ± 0.01	2.89 ± 0.01

The increase of the ceramic nanoinclusions up to 1.0% into the flux continuously decreases the effective diffusion coefficient of the interfacial Cu–Sn IMCs layer showing that this is an effective way to reduce the thickness of the isothermally aged solder joints, which is very important for thin microjoints.

**Fig. 7:**
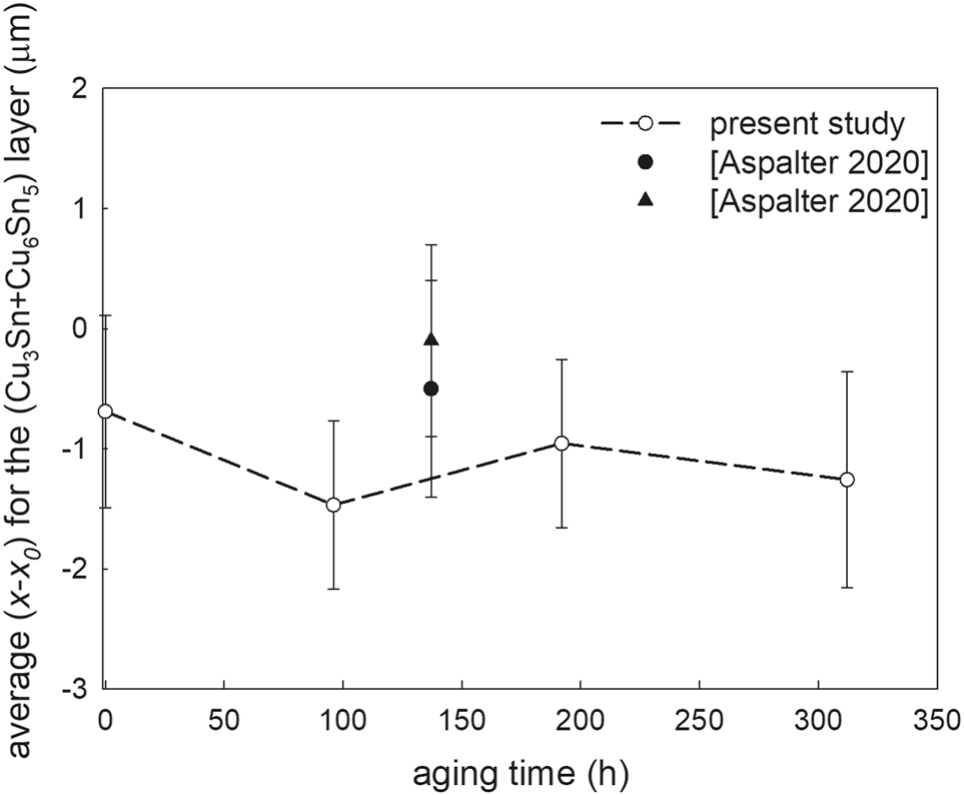
The aging time dependence of the difference in the average thickness of the interfacial IMCs layer in the solder joints with 0.5 wt% ceramic nanoinclusions at 453 K (filled circle—SAC305 + ZrO_2_ NPs; filled square—SAC305 + SiO_2_ NPs)

Unfortunately, we have not found any literature data on the effective diffusion coefficient of the interfacial IMCs layer in both undoped and doped Sn–3.5Ag solder joints. The obtained results are compared with the literature data for the hybrid SAC305 solder joint with ZrO_2_ and SiO_2_ nanoadditions, where these two ceramic powders have shown the most profound effect compared to the other ceramic powders used in that study, namely Al_2_O_3_ NPs and TiO_2_ NPs (Fig. 7). As shown from Fig. 7, the addition of 0.5 wt% of ceramic nanoparticles into the flux decreased the average thickness of the interfacial Cu–Sn IMC layer in the thermally aged Sn-based solder joints, while this effect is more profound in the investigated Sn–3.5Ag solder joint comparing with the literature data for the SAC305 solder joint (Aspalter et al. 2020). This effect remains constant within the entire aging time for the investigated solders supposed that the thermal constraints do not have a significant impact on the adsorbed NPs on the Cu_6_Sn_5_ surface. Furthermore, Fig. 7 shows that the addition of the ZrO_2_ nanoparticles seems to have a more profound effect on the interfacial IMC layer in the thermally aged hybrid SAC305 solder joints than SiO_2_ (Aspalter et al. 2020). However, additional investigations focused on the hybrid Sn–3.5Ag + SiO_2_ joints are needed to confirm it.

## Conclusions

The present study has shown that the minor additions of ZrO_2_ NPs into the flux up to 1.0 wt% leads to a decrease in the growth kinetics of the interfacial Cu–Sn IMCs layer in the Sn–3.5Ag/Cu solder joint. The morphology of the interfacial Cu_6_Sn_5_ IMC layer was transformed from the discontinued scalloped-type shape to the continued one. It is supposed that the nanosized ZrO_2_ inclusions as adsorption elements have changed the driving force for the growth of the interfacial IMCs. The largest decrease in the thickness of the Cu–Sn layer in the as-reflowed hybrid joints has been detected in the samples with 0.2 wt% ZrO_2_, supposing a possible agglomeration of nanoparticles by increasing their amount in the flux. An analysis of the aged samples has shown that the growth rate of the Cu_3_Sn IMC was higher than that of the Cu_6_Sn_5_ IMC at the aging temperatures of 453 K. The calculated effective diffusion coefficient of the aged samples has shown a decrease in the values for the solder joints doped with ZrO_2_ nanoparticles through flux.

## Data Availability

All data generated or analyzed during this study are included in this article.

## Abbreviations

NP: Nano-sized particle
IMC: Intermetallic compound
SEM: Scanning electron microscopy
EDS: Energy-dispersive spectrography
BSE: Back-scattered electron
ICSD: Inorganic Crystal Structural Databas
